# Selenium and Prostate Cancer Prevention: Insights from the Selenium and Vitamin E Cancer Prevention Trial (SELECT)

**DOI:** 10.3390/nu5041122

**Published:** 2013-04-03

**Authors:** Holly L. Nicastro, Barbara K. Dunn

**Affiliations:** 1 Cancer Prevention Fellowship Program, Nutritional Science Research Group, Division of Cancer Prevention, National Cancer Institute, 9609 Medical Center Dr, Rockville, MD 20850, USA; E-Mail: holly.nicastro@nih.gov; 2 Chemoprevention Agent Development Research Group, Division of Cancer Prevention, National Cancer Institute, 9609 Medical Center Dr, Rockville, MD 20850, USA

**Keywords:** selenium, SELECT, prostate cancer, chemoprevention

## Abstract

The Selenium and Vitamin E Cancer Prevention Trial (SELECT) was conducted to assess the efficacy of selenium and vitamin E alone, and in combination, on the incidence of prostate cancer. This randomized, double-blind, placebo-controlled, 2 × 2 factorial design clinical trial found that neither selenium nor vitamin E reduced the incidence of prostate cancer after seven years and that vitamin E was associated with a 17% increased risk of prostate cancer compared to placebo. The null result was surprising given the strong preclinical and clinical evidence suggesting chemopreventive activity of selenium. Potential explanations for the null findings include the agent formulation and dose, the characteristics of the cohort, and the study design. It is likely that only specific subpopulations may benefit from selenium supplementation; therefore, future studies should consider the baseline selenium status of the participants, age of the cohort, and genotype of specific selenoproteins, among other characteristics, in order to determine the activity of selenium in cancer prevention.

## 1. Introduction

Prostate cancer is the second leading cause of cancer death among men in the United States (US) and is the most commonly diagnosed non-cutaneous cancer, with 1 in 6 men expected to be diagnosed with this disease in their lifetimes [1]. In 2012, an estimated 241,740 new cases were diagnosed in the US and approximately 28,170 men died of prostate cancer [2]. While 81% of prostate cancers are diagnosed in the early stage and treated effectively with surgery or radiation, these treatments often result in poorer quality of life due to side effects like incontinence, impotence, or declining bowel function [3,4]. Current treatments for advanced prostate cancer are largely palliative. Many known risk factors for prostate cancer are non-modifiable, including age, race, and genetic factors, whereas modifiable risk factors associated with prostate cancer include obesity, physical activity, and possibly dietary factors [5]. Prostate cancer screening is controversial due to overdiagnosis and overtreatment of non-fatal disease, and the United States Preventive Services Task Force strongly recommends against prostate-specific antigen (PSA) screening for prostate cancer [6]. Because prostate cancer has a long natural history, mainly non-modifiable risk factors, and an incidence rate that far exceeds the mortality rate, a focus on prevention over screening or early detection offers an appealing area of investigation. Goals for prevention strategies should focus on reducing cancer incidence and delaying cancer diagnosis until the individual succumbs to other causes [7].

Because of the established role of androgens in prostate carcinogenesis and the common use of anti-androgenic therapy for treatment of advanced or recurring prostate cancer, the first large prevention trials for prostate cancer targeted androgens. The Prostate Cancer Prevention Trial (PCPT), sponsored by the National Cancer Institute (NCI) and conducted by the Southwest Oncology Group (SWOG), was the first such trial. The aim of PCPT was to determine whether the 5α-reductase inhibitor (5ARI) finasteride would reduce the prevalence of prostate cancer after 7 years of treatment [8]. Because 5α-reductase catalyzes the conversion of testosterone to the more potent androgen dihydrotestosterone and androgens are promoters of prostate carcinogenesis, investigators hypothesized that pharmacological inhibition of this enzyme would reduce prostate cancer prevalence. Men (*n* = 18,882) over the age of 55 without evidence of prostate cancer detected by digital rectal exam (DRE) or prostate-specific antigen (PSA) levels were randomized to receive either 5 mg/day finasteride or placebo for 7 years. Annual DRE and PSA tests were administered and prostate biopsies were recommended for patients with abnormal results. All men without prostate cancer diagnoses at the end of the study were also requested to undergo biopsies; 7551 men agreed to this end-of-study biopsy. After 7 years, the prevalence of prostate cancer was reduced by 24.8% in the finasteride group compared to the control group. However, this promising result was accompanied by a 27% increase in the rate of high-grade prostate cancer (defined as having a Gleason score of 7 to 10) in the finasteride group, dampening enthusiasm for the use of finasteride as a chemopreventive agent. Among the reasons offered to explain this unexpected outcome included detection bias in the finasteride group. Detection bias in the finasteride group was thought to be due to increased sampling density because finasteride reduces the volume of the prostate gland [9].

The Reduction by Dutasteride of Prostate Cancer Events (REDUCE) study was designed to determine the effect of dutasteride on incident prostate cancer. Dutasteride inhibits 5α-reductase types 1 and 2 while finasteride only inhibits type 1. In this four-year multicenter, randomized, double-blind, placebo-controlled, parallel group study, 6729 men were enrolled. In addition to a negative baseline biopsy, inclusion criteria were based on factors that placed these men at high risk of prostate cancer including age, slightly elevated serum PSA levels (2.5 to 10.0 ng/mL), or previous prostate biopsies due to suspected cancer. Participants were randomized to receive either 0.5 mg dutasteride or placebo daily for 6 months. Free and total PSA levels were measured every six months and biopsies were performed after 2 and 4 years or when clinically indicated. Dutasteride was associated with a relative risk reduction of prostate cancer of 22.8%. During the four-year study period, rates of high grade prostate cancer were similar between the dutasteride and the placebo group, though in years 3 and 4, there was a small statistically significant increase in rates of tumors with Gleason scores of 8–10 in the dutasteride group [10]. Due to the concerns about increasing risks of high grade prostate cancer, a US Food and Drug Administration (FDA) advisory panel voted overwhelmingly not to approve finasteride or dutasteride for prostate cancer prevention [11]. 

Concomitant with the interest in anti-androgens, a totally independent approach to prostate cancer chemoprevention involved nutritional agents, specifically vitamin E and selenium. Secondary analyses of other large-scale chemoprevention trials had suggested that these compounds may decrease risk of prostate cancer [12,13]. Further controlled intervention trials, human observational studies, and preclinical studies all provided evidence for potential chemopreventive efficacy of these compounds. Adding to the appeal, both agents are naturally-occurring micronutrients essential to human health that have antioxidant activities. In this review, we will describe the rationale, results, and implications of the Selenium and Vitamin E Cancer Prevention Trial (SELECT).

## 2. Selenium

### 2.1. Dietary Sources and Supplements

Selenium is a nutritionally essential trace mineral. Selenium enters the food chain from the soil in the form of selenate (SeO_4_^2^) or selenite (SeO_3_^−2^) and is converted in plants to organic forms, largely l-selenomethionine and to a lesser extent l-selenocysteine [14,15]. Selenium concentrations in foods can therefore vary widely based on the selenium content of the soil. For example, Ireland, Israel, and the western US have high soil selenium content, while certain regions of China have very low soil selenium content [16]. In fact, Keshan disease, a congestive cardiomyopathy, first observed in Keshan County of Heilongjiang province, Northeast China, was found to be caused by a combination of dietary deficiency of selenium and the presence of a mutated strain of Coxsackievirus [17].

The richest dietary sources of selenium are Brazil nuts, meats, fish, eggs, and cereals. Selenium is also found in lesser amounts in cruciferous vegetables, garlic, and mushrooms [18,19].

Selenium is available in supplement form as selenomethionine or as selenized yeast, yeast grown in a selenium-rich medium. Commercially available selenized yeast can provide up to 1000 to 2000 μg/g selenium, over 90% of which is selenomethionine [20]. Certain selenium supplements, particularly weight loss products or infant formulas, contain sodium selenite or sodium selenate, though these inorganic forms are not highly bioavailable. 

### 2.2. Selenium Metabolism and Biological Activities

**Figure 1:**
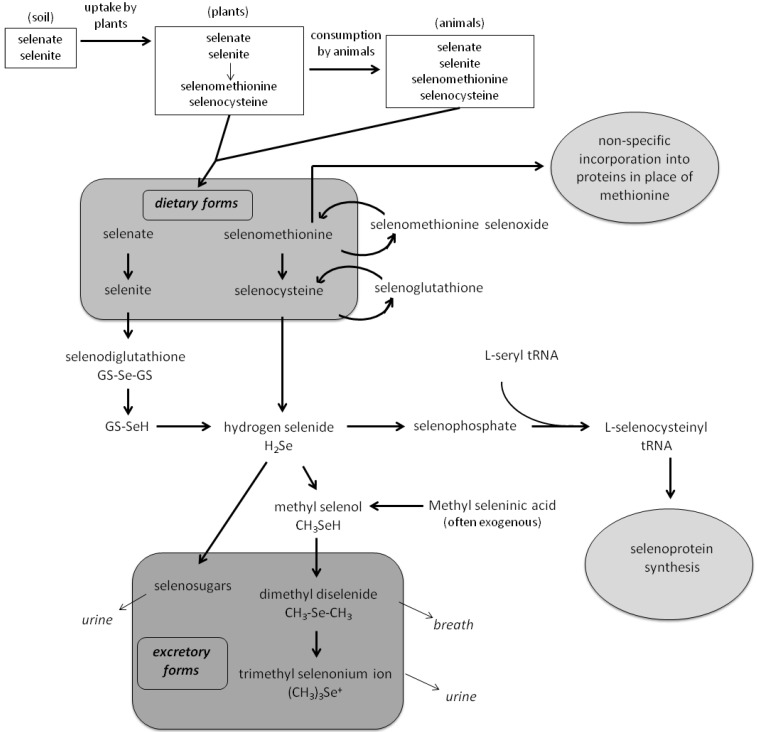
Selenium biology.

Dietary selenium, as selenomethionine, selenocysteine, selenate, or selenite, is essential for selenoprotein synthesis. Selenomethionine can be nonspecifically incorporated into proteins in place of methionine or converted to selenocysteine via a trans-sulfuration pathway. Selenocysteine, either from the diet or derived from selenomethionine, can be converted to hydrogen selenide, a key metabolite integral to both selenocysteine insertion into proteins and selenium excretion. Selenate is reduced to selenite by glutathione, and selenite undergoes further glutathione reduction to hydrogen selenide (Figure 1) [21,22,23]. Therefore, all dietary forms of selenium can be used for selenoprotein synthesis following conversion to hydrogen selenide.

Selenophosphate synthetase converts hydrogen selenide to selenophosphate. Selenophosphate then reacts with l-seryl-tRNA to form l-selenocysteinyl-tRNA, the latter of which is inserted into selenoproteins where specified by the UGA codon in mRNA [24]. Selenocysteine, considered the 21st amino acid, is integral to the activity of selenoproteins [24]. Twenty-five human selenoproteins have been identified, including glutathione peroxidases (GPx), thioredoxin reductases (TR), thyroid hormone deiodinases, selenophosphate synthetase, and several uncharacterized proteins (reviewed in [25]). These selenoproteins play an important role in maintaining redox balance and proper cellular functioning [26].

Hydrogen selenide can also be mono-, di-, or tri-methylated for excretion. Methyl selenol is the major urinary excretory form of selenium. With larger doses of selenium, dimethyl selenium is exhaled from the lungs and the trimethylselenonium ion is excreted in the urine [27]. While the methylation pathway is the main excretion pathway of selenium, selenosugars have also been reported in the urine [28]. The activities of intracellular selenium metabolites including methyl selenol determine the clinical efficacy of selenium, including its chemopreventive effects [29,30,31].

### 2.3. Nutritional Requirements

The recommended dietary allowance (RDA) for selenium for adult men and women is 55 μg/day [32]. This RDA is based on the daily selenium intake necessary for maximal activity of GPx-3. However, with an intake of 55 μg/day, not all selenoproteins’ activity levels would be maximal. Others have suggested that an RDA of 80 μg/day for men is more appropriate for achieving selenium balance [33]. Deficiency symptoms, including loss of immunocompetency, progression of viral infections, and reproductive symptoms, became apparent with intake <11 μg/day [34,35]. Symptoms of selenium toxicity, or selenosis, including hair and nail brittleness/loss, gastrointestinal disturbances, skin rash, garlic breath odor, fatigue, or irritability, appeared with 800 μg/day of selenium intake. Based on this observation and using an “uncertainty factor” of 2, half of 800 μg/day, *i.e.*, 400 μg/day is considered the tolerable upper limit [32].

According to reports using data from the National Health and Nutrition Examination Survey (NHANES) 2003–2006, a nationally representative cross-sectional survey, the usual intake for individuals over the age of 19 was 109 ± 1 μg/day from naturally-occurring dietary sources and 126 ± 1 μg/day from naturally occurring sources plus supplements. Less than 1% of adults had intakes below the estimated average requirements (EAR) and only 0.1% ± 0.4% had intakes above the tolerable upper limit [36].

NHANES data from 2003 to 2006 also show that 19% ± 1% of males reported taking a dietary supplement that contained selenium, including multivitamin-multimineral supplements. Older men were more likely to take any dietary supplements, including those containing selenium. Of men 51–70 years of age and over the age of 70, 30% ± 2% and 32% ± 2% were taking supplemental selenium, respectively [37]. Overall, for all individuals, users of any dietary supplement were more likely to be of non-Hispanic white race, have lower BMI, more education, be less likely to smoke, be more physically active, and consume more fruits and vegetables. For men, but not women, selenium supplement users had higher food selenium intake than non-selenium supplement users, suggesting that those taking supplements were not doing so out of nutritional need. However, selenium supplementation did not affect the percentage of men (0.1% ± 0%) in the analysis who had inadequate selenium intake; in other words, men with already adequate dietary selenium were the same men using selenium supplements [38]. These NHANES analyses suggest that men in the US have adequate selenium intake.

### 2.4. Prostate Cancer Prevention by Selenium—Rationale for SELECT

Although the data are inconsistent, a number of preclinical and epidemiological studies have supported a role for selenium in inhibition of cancers, in some cases including prostate cancer [39,40,41]. In a review of animal studies investigating selenium’s chemopreventive efficacy, Combs and Gray noted that selenium significantly reduced tumor incidence in two-thirds of the studies and many of these studies showed tumor reductions of 50% or more in a variety of non-prostate cancer tumor sites [22].

Laboratory studies have been especially useful in highlighting potential antitumorigenic mechanisms of selenium. These studies have been reviewed in depth elsewhere [22,29,34]. Cancer-relevant cellular pathways and physiological processes affected by selenium include carcinogen bioactivation, cell proliferation, apoptosis, and immune function. Many preclinical studies testing the chemopreventive efficacy and mechanisms of selenium have used inorganic selenium in the form of selenite. 

Very few *in vivo* prostate cancer prevention studies that used tumor endpoints have been conducted in animal models (Table 1). Two studies with designs relevant to SELECT tested the efficacy of l-selenomethionine or dl-α-tocopherol on prostate cancer incidence and multiplicity and reported null findings [42,43]. Other *in vivo* prevention studies either only used selenium in combination with other agents and not alone or used methylated or inorganic selenium [44,45,46,47]. Despite the null findings in animal studies, epidemiological studies and analyses of data on secondary endpoints in clinical trials provided evidence for potential effects of selenium.

#### 2.4.1. Epidemiological Studies

The first epidemiological evidence for cancer preventive activities of selenium came from ecological analyses that suggested an association between higher risk and mortality of certain cancers in selenium-deficient regions of the US compared to selenium-replete areas [48,49]. Numerous case-control studies have found that a general trend exists between higher selenium levels (as assessed by pre-diagnostic blood levels, serum levels, toenail selenium levels, or dietary selenium intake) and decreased cancer incidence and mortality (reviewed in [22,34,50]).

**Table 1:** *In vivo* animal studies that assessed prostate cancer prevention by selenium.

Animal Model [Ref.]	Agent and Dose	Results
*Studies relevant to SELECT trial design*		
*N*-Nitroso-*N*-methylurea (MNU) + testosterone-treated Wistar-Unilever rats [42]	l-selenomethionine (1.5 or 3 mg/kg diet) dl-α-tocopherol (4000 or 2000 mg/kg diet) l-selenomethionine ( 3 mg/kg diet ) + dl-α-tocopherol (2000 or 5000 mg/kg diet) Selenized yeast (target Se levels of 9 or 3 mg/kg diet) Control	No effect on prostate cancer incidence in any group
Testosterone + estradiol-treated NBL rat [43]	l-selenomethionine (1.5 or 3.0 mg/kg diet) dl-α-tocopherol (4000 or 2000 mg/kg diet) Control	No effect on prostate tumor incidence, multiplicity, or death in any group
*Studies on selenium in combination with other agents only*		
*Lady* transgenic mice [44]	α-tocopherol succinate (800 IU) + l-selenomethionine (200 μg) + lycopene (50 mg) α-tocopherol succinate (800 IU) + l-selenomethionine (200 μg) Control	Increased survival ( *p* < 0.0001) in both treatment groups compared to control, no effect on prostate tumor incidence in any group
*Lady* transgenic mice [45]	α-tocopherol succinate (800 IU) + l-selenomethionine (200 μg) + lycopene (50 mg) Control	Four-fold decrease in prostate cancer incidence in animals treated with 3 agents combined compared to control animals ( *p* < 0.0001)
*Studies on other forms of selenium*		
Transgenic adenocarcinoma mouse prostate (TRAMP) model [46]	Methylseleninic acid (3 mg selenium/kg body weight) for 10 weeks Methylseleninic acid (3 mg selenium/kg body weight) for 16 weeks Control	Decreased cancer-specific mortality in methylseleninic acid groups compared to control group ( *p*_10__ weeks_ = 0.0078, *p*_16__ weeks_ = 0.0385)
MNU + testosterone-treated Wistar rats [47]	Sodium selenite (4 mg/L in drinking water/day) Control	No effect on prostate intraepithelial neoplasia, decreased prostate cancer multiplicity by 44.6% in sodium selenite group compared to control group

Studies of the association of selenium and prostate cancer specifically support this same general association [34,50,51]. In a matched case-control study nested within the prospective cohort Health Professionals Follow-Up Study, the highest quintile of toenail selenium level was associated with a decreased risk of advanced prostate cancer compared to the lowest quintile (OR: 0.49, 95% CI: 0.25–0.96, *p*-trend = 0.11) [52]. This decreased risk was more pronounced after adjustment for known prostate cancer risk factors including family history and body mass index (OR: 0.35, 95% CI: 0.16–0.78, *p*-trend = 0.03). However, this association was not observed in two nested case-control studies among men in the European Prospective Investigation into Cancer and Nutrition (EPIC) cohort. The investigators found that plasma selenium was not associated with prostate cancer risk. Reasons for the discrepancies in findings are unclear, but the authors noted that the European cohort had substantially lower plasma selenium concentrations (mean: 70 μg/L) than those found in men in the US (>100 μg/L). Therefore, it is possible that the selenium levels of men in even the highest quintile in the EPIC cohort were below levels necessary for cancer prevention.

The World Cancer Research Fund/American Institute for Cancer Research (WCRF/AICR) reviewed 17 cohort studies, 3 ecological studies, 14 case-control studies, and 1 clinical trial (NPC, see below) and concluded that selenium and foods containing selenium probably protect against prostate cancer due to dose-response relationships and evidence for plausible mechanisms [53]. A meta-analysis in this report indicated that there was a 5% decrease in the risk of prostate cancer and a 13% decrease in the risk of advanced or aggressive prostate cancer for every 10 ng/mL increase in plasma selenium or a 20% decrease in the risk of advanced or aggressive prostate cancer for every 100 ng/g increase in toenail selenium. While this report was released after the design and implementation of SELECT and the report focused on the rationale and implications of SELECT, many of the studies used in the review and meta-analysis were published prior to the start of the trial. Further, the findings of this report are important to the future of selenium and cancer prevention.

#### 2.4.2. Clinical Trials

Large randomized trials in Qidong and Linxian, China, were among the first to demonstrate cancer preventive activities of selenium in humans, though these trials investigated liver, gastric, and esophageal, and not prostate, cancers. In a community intervention trial in Qidong, a selenium-deficient region, salt supplemented with sodium selenite, which provided 50–80 μg of selenium per day, decreased the incidence of primary liver cancer by 35.1% over 8 years in regions receiving the salt compared to control regions [54]. When the intervention ended and the selenized salt was no longer administered, incidence of primary liver cancer increased in the intervention regions. A concurrent clinical trial in the same region among 226 hepatitis B surface antigen positive participants showed that 200 μg selenium as selenized yeast daily for 4 years resulted in no cases of primary liver cancer among 113 individuals compared to 7/113 new cases in the placebo group [54].

The Linxian Nutritional Intervention Trials aimed to determine whether supplementation with multiple vitamins and minerals would decrease the risk of esophageal and gastric cardia cancers among those with esophageal dysplasia. In the first trial, 29,854 participants 40–69 years old with esophageal dysplasia were recruited and randomized to receive one of eight combinations of vitamins and minerals. Among participants receiving Factor D, which consisted of 50 μg selenium, 30 mg vitamin E, and 15 mg β-carotene daily, cancer mortality decreased by 13% and stomach cancer mortality decreased by 21% over 5 years compared to those not receiving Factor D [55]. In the second trial, 3000 individuals with esophageal dysplasia were randomized to receive either a multivitamin/multimineral supplement containing 12 micronutrients including 50 μg selenium and a separate 15 mg β-carotene supplement daily, or two placebos. After 6 years of follow-up, total cancer mortality decreased by 4%, gastric/esophageal cancer mortality decreased by 8%, and esophageal cancer mortality decreased by 16% in the supplement group compared to the placebo group; however, none of these decreases was statistically significant and cancer incidence rates were similar between the two groups [56].

The primary hypothesis-generating trial that prompted the use of selenium in SELECT was the Nutritional Prevention of Cancer (NPC) Trial [13]. This randomized, double-blind, placebo-controlled trial enrolled 1312 participants from the eastern US who had a history of skin cancer. The aim of the study was to test the efficacy of selenium supplementation on preventing non-melanoma skin cancer. Secondary endpoints included total cancer incidence, total cancer mortality, and incidence and mortality of lung, prostate, and colorectal cancers. Participants were randomized to receive either 200 μg of selenium in the form of selenized yeast or a placebo daily. After 8271 person-years of follow-up, the primary endpoint of skin cancer was not favorably affected by the selenium intervention. However, an observed decrease in other cancer incidence and mortality became evident. Prostate cancer was decreased in the selenium group by 64% after 4.5 years and by 49% after 10 years (incidence RR: 0.51, 95% CI: 0.29–0.87). This decrease was most pronounced in former smokers. Stratified analysis showed that selenium supplementation lowered the incidence of prostate cancer in those in the lowest two tertiles of baseline selenium status (RR_tert1_: 0.14, 95% CI: 0.02–0.59; RR_tert2_: 0.39, 95% CI: 0.14–0.99), but not in those in the highest tertile of baseline selenium status (RR: 1.20, 95% CI: 0.50–2.97). A significant interaction between treatment group and baseline selenium status was observed. The risk reduction for prostate cancer was also confined to those men who had baseline PSA levels ≤ 4.0 ng/mL (RR: 0.35, 95% CI: 0.13–0.87), although no significant interaction was observed between treatment and baseline PSA [57]. 

Following the NPC trial, a small pilot study was initiated to test the effect of selenium supplementation on selenium levels in the prostate and seminal vesicles [58]. Men with organ-confined prostate cancer (*n* = 66) who were planning on surgical resection of the prostate were randomized to receive either 200 μg l-selenomethionine daily for 14–31 days or standard of care observation (control group) during the pre-surgical period. Prostate selenium concentrations were 22% higher in the selenium group than in the control group while selenium concentrations in the seminal vesicles were similar in both groups. This study demonstrated that supplemental selenium selectively accumulated in the prostate, providing additional biological plausibility for chemopreventive effects of selenium against prostate cancer.

The Alpha-Tocopherol, β-Carotene Cancer Prevention (ATBC) study also served as a hypothesis-generating trial for SELECT. In this randomized, double-blind, placebo-controlled trial of 29,133 male smokers aged 50–69 at enrollment, the primary endpoint of lung cancer incidence was not affected by alpha-tocopherol intake. However, as a secondary endpoint, the 50 mg/day alpha-tocopherol decreased the incidence of prostate cancer from 17.8/10,000 person-years in the placebo group to 11.7 in the α-tocopherol group after 5–8 years of follow-up [59]. Preclinical evidence, as well as findings from the Linxian trials, suggested that the combination of selenium and vitamin E might offer additional protection for prostate cancer prevention.

## 3. SELECT

### 3.1. Rationale and Objectives

In light of findings in the NPC and ATBC trials, the Selenium and Vitamin E Cancer Prevention Trial (SELECT) was funded by the NCI and implemented by SWOG. SELECT represented the second phase III NCI-sponsored prostate cancer prevention trial. 

The primary objective of SELECT was to assess the efficacy of selenium and vitamin E alone and in combination on the incidence of prostate cancer. Pre-specified secondary endpoints included prostate cancer-free survival, all cause mortality, incidence and mortality of other cancer types including lung and colorectal cancers, overall cancer incidence and survival, and disease potentially impacted by chronic administration of selenium and/or vitamin E. Investigators also aimed to monitor serious cardiovascular events, assess quality of life, study serum micronutrient levels and prostate cancer risk, and evaluate biological and genetic markers associated with the risk of prostate cancer.

### 3.2. Agent Formulation and Dose

Despite the use of selenized yeast in the NPC trial [13], l-selenomethionine was chosen for SELECT, based on the advice of an NCI-sponsored panel of experts. This recommendation was founded on large batch-to-batch variability and lack of commercial availability of selenized yeast. Further, laboratory analyses had determined that l-selenomethionine was the predominant selenium species in selenized yeast available at the time. The daily dose of 200 μg was similar to the dose of 200 μg selenized yeast used in the NPC trial [13]; however the precise amount of selenium delivered by the selenized yeast was highly variable [60], so it is difficult to directly compare the doses between the two trials except to say that 200 μg l-selenomethionine delivers more selenium than 200 μg selenized yeast.

The racemic mix of α-tocopherol, which includes the d- and l-isomers, was chosen based on the association of long-term supplementation with this formulation with reduced prostate cancer incidence in the ATBC trial. The dose of 400 mg/day was chosen based on its use in vitamin supplements (suggesting safety) and its potential benefits for non-cancer diseases, including cardiovascular disease and Alzheimer’s disease. However, this dose was eight times higher than that used in the ATBC study, 50 mg/day [59].

### 3.3. Trial Design and Outcome Ascertainment

SELECT was a prospective, randomized, double-blind, placebo-controlled 2 × 2 factorial design clinical trial of selenium and vitamin E alone and in combination in eligible healthy men who were at elevated risk by virtue of age and/or African ancestry. Participants were randomized to receive daily oral doses of either 200 μg selenium plus placebo, 400 mg α-tocopherol plus placebo, 200 μg selenium plus 400 mg α-tocopherol, or two placebos. The planned duration of the study was 12 years with a 5-year accrual period, and 7–12 years of intervention.

A sample size of 32,400 men was required to address five predetermined comparisons: vitamin E *vs.* placebo, selenium *vs.* placebo, vitamin E plus selenium *vs.* placebo, vitamin E plus selenium *vs.* vitamin E alone, and vitamin E plus selenium *vs.* selenium alone. This sample size provided adequate power to detect ≥25% decreases in the incidence of prostate cancer for selenium or vitamin E alone and an additional 25% decrease for selenium and vitamin E combined compared to either agent alone.

Prostate cancer was assessed based on a recommended routine clinical diagnostic evaluation, including yearly DRE and serum PSA measurement. Prostate biopsies were performed at the discretion of study physicians, with additional study recommendations of biopsy for participants with DRE suspicious for cancer or elevated PSA. No end-of-study biopsies were required. 

### 3.4. Recruitment, Enrollment, Cohort, and Baseline Characteristics

Eligibility was based on elevated risk of prostate cancer due to age. Caucasian men ≥55 years old and African American men ≥50 years old were targeted. Other inclusion criteria required men to be healthy, have total PSA ≤ 4.0 ng/mL, have a DRE not suspicious for cancer, have no previous prostate cancer or high grade prostate intraepithelial neoplasia, have normal blood pressure, not be currently taking anticoagulation therapy, and be willing to stop taking off-study supplements [61]. A total of 35,533 eligible men from the US, Canada, and Puerto Rico were enrolled in a 3-year period, exceeding the goals for both number of participants and for length of the accrual period. The cohort included 21% minorities (12% African American, 7% Hispanic, and 2% other) [62]. Prostate cancer risk factors, including age, race, education level, baseline PSA, and smoking status were equally balanced among the four treatment groups following randomization [62].

### 3.5. Primary Endpoint Results

Results from the trial were released in two reports [62,63]. An independent data and safety monitoring committee unanimously decided after 7 years of the planned 12-year study that supplement use should be discontinued due to lack of evidence of benefit. The first report of SELECT results included data current as of 23 October 2008, the date on which study sites were advised to discontinue supplement administration. The median follow-up time was 5.46 years with a range of 4.17–7.33 years. Rates of prostate cancer did not differ significantly among the four intervention arms. The hazard ratio (HR) of prostate cancer was 1.13 (99% CI: 0.95–1.35) for the vitamin E group, 1.04 (99% CI: 0.87–1.24) for the selenium group, and 1.05 (99% CI: 0.88–1.25) for the selenium + vitamin E group, relative to the placebo group (Table 2).

**Table 2:** Primary endpoint results from SELECT, first and second reports [62,63].

	First Report, October 2008				Second Report, July 2011			
	Placebo (*n* = 8696)	Vitamin E (*n* = 8737)	Selenium (*n* = 8752)	Selenium + Vitamin E (*n* = 8703)	Placebo (*n* = 8696)	Vitamin E (*n* = 8737)	Selenium (*n* = 8752)	Selenium + Vitamin E (*n* = 8702)
**Prostate cancer**								
**No. events**	416	473	432	437	529	620	575	555
**HR (99% CI)**	1 (reference)	1.13 (0.95–1.35)	1.04 (0.87–1.24)	1.05 (0.88–1.25)	1 (reference)	1.17 (1.004–1.36) ^a^	1.09 (0.93–1.27)	1.05 (0.89–1.22)
**Method of diagnosis, *n* (%)**								
**Prostate biopsy**	404 (97)	458 (97)	419 (97)	420 (96)	n.r. ^b^	n.r.	n.r.	n.r.
**Other/unknown**	12 (3)	15 (3)	13 (3)	17 (4)	n.r.	n.r.	n.r.	n.r.
**Gleason score, *n* (%)**								
**2–6**	240 (66)	249 (63)	217 (60)	220 (60)				
**4–6**					286 (69)	310 (67)	281 (64)	281 (63)
**7**	101 (28)	124 (31)	124 (34)	115 (32)	102 (24)	118 (25)	135 (31)	124 (28)
**8–10**	24 (7)	23 (6)	20 (6)	30 (8)	31 (7)	37 (8)	26 (6)	40 (9)
**Not graded**	51	77	71	72	110	155	133	110

^a^ *p* = 0.008. ^b^ n.r.: not reported.

**Figure 2:**
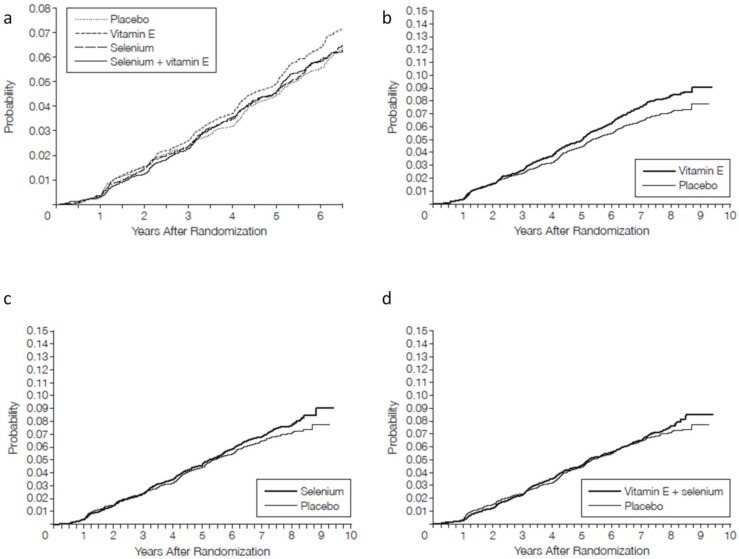
Cumulative incidence of prostate cancer from both SELECT reports (**a**) Results from the 2009 initial report. Median follow-up time was 5.46 years. (**b**–**d**) Results from the 2011 report including data on an additional 54,464 person-years of follow-up; (**b**) Vitamin E *vs.* placebo; (**c**) Selenium *vs.* placebo; (**d**) Vitamin E + selenium *vs.* placebo. Adapted from [62,63] with permission.

The second report included data current as of July 2011, which contained an additional 54,464 person-years of follow up since the first report. An additional 521 prostate cancers were diagnosed, 12113 in the placebo group, 147 in the vitamin E group, 143 in the selenium group, and 118 in the selenium + vitamin E group. The rates of prostate cancer detection in the selenium and selenium + vitamin E groups did not differ significantly from the rate in the placebo group. However, risk of prostate cancer in the vitamin E group was increased 17% compared to the placebo group (HR = 1.17, 99% CI: 1.004–1.36, *p* = 0.008). The 13% increase in risk seen in the vitamin E group in the first report, while not statistically significant, suggested that the later significant 17% increase in risk was not an outlier. Further, the graph of cumulative incidence of prostate cancer by supplement group suggests that the vitamin E group curve begins to diverge from the placebo curve by about 3–4 years after randomization (Figure 2). There was no increased risk of prostate cancer for the vitamin E + selenium group.

The majority of prostate cancers were diagnosed by prostate biopsy “for cause” due to increased PSA levels or abnormal DRE results. Most were early stage and low Gleason grade. Importantly, stage and grade did not differ by treatment group. In addition, when the entire study population was examined for PSA levels, levels did not differ by treatment group.

### 3.6. Secondary Endpoints and Adverse Outcomes

Secondary endpoints specified *a priori* included other cancers, including colorectal and lung cancer, total cancer incidence, cardiovascular events, diabetes, and deaths. There were no significant differences among treatment groups for any of these endpoints (Table 2). Type 2 diabetes mellitus was of particular interest because previous reports had linked higher prevalence with higher selenium levels and higher incidence with long-term selenium supplementation [64,65,66]. A somewhat, though non-significantly, increased risk of type 2 diabetes mellitus in the selenium arm (HR: 1.07; 99% CI: 0.94–1.22) was observed in the first report. However, this slight increase was diminished in the second report (HR: 1.04; 99% CI: 0.93–1.17), suggesting that selenium’s possible link with diabetes was less concerning than previously thought.

### 3.7. Adherence to Study Supplements

Adherence to study supplements was assessed by pill counting and participant diaries. Serum levels of selenium (Table 3) and cholesterol-adjusted α-tocopherol and γ-tocopherol levels were also measured in a bioadherence subcohort. On average, adherence by pill count was 83% at year 1 and 65% at year 5. Serum selenium and α-tocopherol levels rose only in participants randomized to receive those agents and not in the other groups, demonstrating good adherence and minimal drop-ins. The drop-in rate was also assessed via asking participants whether they took either supplement; rates were 3.1% or less for vitamin E and 1.8% or less for selenium [62].

### 3.8. Follow-Up

Following the discontinuation of supplement use and release of the primary data, SELECT transitioned into an observational cohort study, the SELECT Centralized Follow-up Study (SELECT CFU). As of December 2011, 17,761 participants, 58% of the 32,569 SELECT participants who were still alive and not refusing further contact, had enrolled in SELECT CFU [67]. In addition, four ancillary studies evaluating the effect of selenium and vitamin E on colon cancer screening procedures, memory changes, lung function, and age-related macular degeneration are ongoing. 

**Table 3:** Secondary endpoints and adverse outcomes in SELECT by report date [62,63].

	First Report, October 2008				Second Report, July 2011			
Trial Arm	Placebo (*n* = 8696)	Vitamin E (*n* = 8737)	Selenium (*n* = 8752)	Selenium + Vitamin E (*n* = 8703)	Placebo (*n* = 8696)	Vitamin E (*n* = 8737)	Selenium (*n* = 8752)	Selenium + Vitamin E (*n* = 8702)
**Any cancer, including prostate ^a^**								
**No. events**	824	856	837	846	1108	1190	1132	1149
**HR (99% CI)**	1 (reference)	1.03 (0.91–1.17)	1.01 (0.89–1.15)	1.02 (0.90–1.16)	1 (reference)	1.07 (0.96–1.19)	1.02 (0.92–1.14)	1.02 (0.92–1.12)
**Lung cancer**								
**No. events**	67	67	75	78	92	104	94	104
**HR (99% CI)**	1 (reference)	1.00 (0.64–1.55)	1.12 (0.73–1.72)	1.16 (0.76–1.78)	1 (reference)	1.11 (0.76–1.61)	1.02 (0.70–1.50)	1.11 (0.76–1.62)
**Colorectal cancer**								
**No. events**	60	66	63	77	75	85	74	93
**HR (99% CI)**	1 (reference)	1.09 (0.69–1.73)	1.05 (0.66–1.67)	1.28 (0.82–2.00)	1 (reference)	1.09 (0.72–1.64)	0.96 (0.63–1.46)	1.21 (0.81–1.81)
**Other primary cancer**								
**No. events**	306	274	292	290	579	570	557	594
**HR (99% CI)**	1 (reference)	0.89 (0.72–1.10)	0.95 (0.77–1.17)	0.94 (0.76–1.16)	1 (reference)	0.97 (0.83–1.14)	0.96 (0.83–1.13)	1.02 (0.92–1.14)
**Diabetes**								
**No. events**	669	700	724	660	869	918	913	875
**HR (99% CI)**	1 (reference)	1.04 (0.91–1.18)	1.07 (0.94–1.22)	0.97 (0.85–1.11)	1 (reference)	1.05 (0.93–1.17)	1.04 (0.93–1.17)	0.99 (0.89–1.12)
**Any cardiovascular event**								
**No. events**	1050	1034	1080	1041	n.r. ^b^	n.r.	n.r.	n.r.
**HR (99% CI)**	1 (reference)	0.98 (0.88–1.09)	1.02 (0.92–1.13)	0.99 (0.89–1.10)				
**Cardiovascular events, grade ≥4**								
**No. events**	n.r.	n.r.	n.r.	n.r.	969	909	939	943
**HR (99% CI)**					1 (reference)	0.93 (0.83–1.05)	0.97 (0.86–1.09)	0.97 (0.86–1.09)
**Deaths, all cause**								
**No. events**	382	358	378	359	564	571	551	542
**HR (99% CI)**	1 (reference)	0.93 (0.77–1.13)	0.99 (0.82–1.19)	0.94 (0.77–1.13)	1 (reference)	1.01 (0.86–1.17)	0.98 (0.84–1.14)	0.96 (0.82–1.12)
**Bioadherence Trial arm**	Placebo (*n* = 285)	Vitamin E (*n* = 290)	Selenium (*n* = 277)	Selenium + Vitamin E (*n* = 257)				
**Serum selenium, μg/L**								
**Baseline, mean**	137.6	135.9	135.0	136.4	n.r.	n.r.	n.r.	n.r.
**IQR**	124.7–151.8	122.4–148.4	123.4–145.9	122.9–150.0				
**6-months visit, mean**	137.4	138.4	223.4	227.0				
**IQR**	123.3–152.0	124.1–154.0	198.6–251.8	199.4–251.2				
**1st annual visit, mean**	138.1	137.7	232.4	228.5				
**IQR**	125.2–152.2	120.1–139.9	204.2–261.4	205.5–258.1				
**2nd annual visit, mean**	132.0	129.8	228.0	220.7				
**IQR**	120.8–143.1	126.2–158.6	206.3–256.9	194.0–249.5				
**4th annual visit, mean**	140.1	143.8	251.6	253.1				
**IQR**	124.3–150.8	126.2–158.6	218.7–275.0	210.5–283.0				

^a^ Numbers for specific types of cancers (lung, colorectal, other primary cancers, prostate cancers from Table 2) may not sum to number of individuals with cancer (described as “any cancer”) due to multiple cancers per person. ^b^ n.r.: not reported.

## 4. Discussion

The results of SELECT showed that neither selenium nor vitamin E alone or in combination decreased the incidence of prostate cancer and that vitamin E supplementation significantly increased the incidence of prostate cancer among healthy men. These findings were surprising given the strong epidemiological findings and the promising results of the hypothesis-generating NPC and ATBC trials. Numerous hypotheses have been generated to explain the null findings. These hypotheses center on the agent formulation and dose that were chosen, the cohort, and the study design.

### 4.1. Agent: Selenium Formulation and Dose

A daily dose of 200 μg of l-selenomethionine was used in SELECT. This is the same dose but a different formulation than the selenized yeast used in the NPC trial. The dose was chosen by an expert panel for NPC based on efficacy and safety data from preclinical studies. However, it is important to note that an optimal dose of selenium for cancer prevention has not been established. It is likely that a narrow range of optimal doses exist, and these doses may depend on the baseline selenium status of the individual.

The use of pure l-selenomethionine is more controversial than the dose chosen. Inorganic selenium had been shown to have better *in vitro* anticancer activities, but these forms were linked to DNA single strand breaks [68]. l-selenomethionine was chosen over selenized yeast largely for logistical reasons, including lack of widely available selenized yeast and batch-to-batch variability in that which was commercially available. While l-selenomethionine is the predominant selenocompound in selenized yeast, the yeast contains numerous other selenocompounds with varying chemopreventive efficacy [69]. Ip and colleagues demonstrated that methylseleninic acid, a metabolite of methyl selenol, is important for the chemopreventive effects of selenium [70]. Selenomethionine can be converted to methyl selenol, but is also non-specifically incorporated into proteins in place of methionine, diverting the selenium away from its active chemopreventive form (Figure 1). Other selenocompounds like selenocysteine, selenite, or selenate, are not used non-specifically in proteins and therefore are more likely to be converted to the potentially antitumorigenic metabolite methyl selenol [23]. However, recent findings by Waters *et al.* indicated that selenomethionine and selenized yeast have similar biological activities in prostatic tissue of dogs [71]. After seven months of supplementation with either selenomethionine or selenized yeast, there were no differences in levels of intraprostatic dihydrotestosterone and testosterone, dihydrotestosterone: testosterone ratio, DNA damage, proliferation, or apoptosis in the prostates of dogs by formulation received. Additionally, the investigators found no differences in toenail or intraprostatic selenium levels by formulation. In light of these results, it seems unlikely that the formulation of selenium used in SELECT is a major reason for the null results observed. However, it is important to compare these formulations in humans. We are aware of one ongoing clinical trial to compare the effect of selenized yeast and selenomethionine on PSA levels and other prostate cancer biomarkers in healthy men [72].

### 4.2. Cohort: Baseline Selenium Status, Age, Genetics

The characteristics of the men recruited, including baseline selenium status, age, and genetics, reflect potentially important differences between NPC and SELECT, and are likely reasons for the null results in SELECT.

#### 4.2.1. Baseline Selenium Status

NPC recruited men from eastern coastal areas of the USA where environmental selenium is low, whereas SELECT recruited US/Canada-wide. This resulted in large discrepancies in baseline selenium status between men in the two trials. The mean baseline serum selenium concentration in NPC was 114 ng/mL; the mean baseline concentration among men in SELECT was 135 ng/mL. In fact, 78% of the volunteers in SELECT had serum levels that were above the lower two tertiles in NPC and therefore above levels of those who saw benefit from selenium in NPC [73].

Waters and colleagues demonstrated that selenium status as determined by toenail selenium concentration exhibits a U-shaped relationship with DNA damage in the prostate in elderly dogs and that this relationship parallels results from human studies [74]. They estimated that the optimal range of toenail selenium levels for prostate cancer risk reduction is 0.80–0.92 ppm, which corresponds to 119–137 ng/mL [75]. Above this level, additional selenium would offer no added benefit and may even be harmful. This estimate is consistent with results from NPC where protection was only seen in men with baseline serum selenium concentrations <123.2 ng/mL [57]. Stratified analysis of SELECT data by quantile baseline selenium status may identify a subset of men who received a benefit from selenium, though it is possible that the baseline selenium status of men in the lowest quantile in this cohort may be above levels at which supplemental selenium could offer protection. 

It is likely that the importance of baseline selenium status was not adequately recognized when SELECT was in its planning stage, and low baseline selenium status was not considered as a criterion for study enrollment nor was a soil selenium-deficient region considered for the study location. Regardless, because SELECT was a very large trial it was necessary to be inclusive with regards to geography in order to accrue an adequate number of participants and the study was conducted through more than 400 clinical sites across the United States, Puerto Rico, and Canada. Further, due to increasing fortification of the food system with selenium in the years since implementation of the NPC trial, it would be difficult to ensure low baseline selenium status by limiting the catchment area to a low soil selenium area.

#### 4.2.2. Genetics

While not a focus of NPC or SELECT, genetics may affect selenium status or an individual’s response to selenium supplementation. In particular, polymorphisms in the genes that encode selenoproteins or proteins involved in selenium metabolism may influence health outcomes. For example, Li *et al.* [76] reported that among men with the AA genotype of codon 16 (rs4880) of *SOD2,* a gene that encodes the mitochondrial antioxidant enzyme manganese superoxide dismutase, men with higher selenium levels had lower risk of total prostate cancer (RR: 0.3, 95% CI: 0.2–0.7) and of clinically aggressive prostate cancer (RR: 0.2, 95% CI: 0.1–0.5) compared to those with lower selenium levels. This protection was much weaker in men with VV or VA genotypes [76]. In an analysis of prostate cancer mortality from the Physicians’ Health Study, three different polymorphisms in the selenoprotein gene *SEP15* (rs479341, rs1407131, and rs561104) significantly affected survival time in men with prostate cancer, either increasing or decreasing survival depending on the polymorphism and the genotype. Further, an interaction exists between selenium and genotype of rs561104. High levels of selenium were associated with decreased prostate cancer mortality only in those with the increased risk homozygous variant genotype and not in those with the wild-type genotype (*P*_interaction_ = 0.02) [77]. The genotype of *GPX1,* which encodes the selenoprotein GPx1, was recently shown to be a determinant of selenium requirements. In a study of 161 men and women, those with the *GPX1* 679 (rs1050450) T/T genotype had significantly lower plasma selenium levels than those with the C/C genotype [78]. The mean plasma selenium level in this study was 142.0 ng/mL, slightly above that of men in SELECT. 

The genotype of these and other genes is expected to contribute to selenium balance and selenium-dependent health outcomes. These findings have implications for future studies on selenium status and supplementation. Stratification of SELECT participants according to allelic status in these relevant genes may elicit relationships between selenium supplementation and prostate cancer risk that were not evident in the trial population as a whole.

#### 4.2.3. Age

The age of the study population is also of interest. Given the long natural history of prostate cancer, it is possible that intervening in men older than 50 years of age may miss a critical window for intervention. For example, in a case-control study addressing breast cancer, another hormonal cancer, an inverse association was observed between soy food intake in adolescents and breast cancer as an adult, but protection was not observed when soy food intake began later in life [79]. Rodent studies have shown that selenite and other selenium metabolites inhibit carcinogen-induced mammary cancer at the stage of initiation by decreasing DNA damage, but that selenium also has post-initiation activities and is most efficacious when administered continuously beginning during initiation [22]. A better understanding of selenium biology and the process and timing for which selenium influences prostate carcinogenesis might better predict the optimal age range at which selenium supplementation should take place. However, in light of the NPC results, intervention in men over the age of 50 still has the potential to yield some anti-cancer benefit.

### 4.3. Design

An important difference between the NPC trial and SELECT is that prostate cancer incidence was a secondary outcome measure of NPC and a primary outcome measure of SELECT. Each trial was adequately powered to detect differences of predetermined magnitude in their respective primary outcomes. In a clinical trial with multiple outcomes, *a prioiri* designation of a primary endpoint protects that measure from concerns of the observed result being due to chance as a result of multiple testing [80]. Secondary endpoints remain at risk of false positive results because of multiple testing. In NPC, results on skin cancer were protected while observations on secondary endpoints, including prostate cancer, were not. Results of the NPC trial were particularly vulnerable to chance findings due to the small sample size of 64 prostate cancer cases in 1312 participants. Therefore, follow-up of the prostate cancer incidence findings from NPC in a large randomized trial where prostate cancer incidence was the primary outcome of interest was essential. Further, in the NPC trial, participants with a history of skin cancer were recruited. Because the primary endpoint of that trial was skin cancer incidence, risk factors for prostate cancer including PSA were not considered for eligibility or exclusion criteria.

SELECT was designed to test the effects of selenium and vitamin E on prostate cancer after 7–12 years of supplementation. It remains a possibility that a lag to effect occurred and that the benefit, or harm, of selenium supplementation is only evident after a much longer period of time, as was the case for tamoxifen in the Royal Marsden Hospital breast cancer chemoprevention trial [81]. Results from the SELECT-CFU cohort study can help to address this issue. 

Additionally, SELECT was designed to test the effects of these agents in a diverse cohort of older men and was not powered to address subgroup analyses. Regardless, findings according to quantile of baseline selenium status, genotype, or other risk factors will be enlightening and may help generate hypotheses for future trials in cohorts that may receive benefit from selenium.

### 4.4. Does Selenium Really Prevent Prostate Cancer?

A number of potential explanations for the failure of selenium and vitamin E individually and together to reduce prostate cancer incidence have been proposed above. However, one possibility is that neither bioactive food component actually has a potent preventive effect on this disease and that the supportive data from randomized trials in humans merely reflected secondary endpoints that did not carry statistical validity. In fact, early laboratory data mimicking the design of what would eventually become the SELECT trial showed no statistically significant reductions in prostate cancer incidence with either selenium (l-selenomethionie) or vitamin E alone or together [42]. Nevertheless, interest remains in pursuing selenium as a potential cancer preventive agent for prostate as well as other cancers.

## 5. Future Directions

Hatfield and Gladyshev proposed that an additional important outcome of SELECT was the need to better understand selenium biology. [23] A clearer view of selenium’s biological activities will aid researchers in choosing the appropriate doses and formulations of future agents. Work is continuing on selenium and its ability to antagonize carcinogenesis. One promising avenue is the ongoing characterization of selenium’s anti-DNA damage activities. The doses and conditions necessary as well as the mechanisms of action may be enlightening [75,82]. Furthermore, many of the 25 selenoproteins identified in humans remain largely uncharacterized regarding tissue specificity, function, regulation, and enzyme kinetics [25]. Ongoing work to characterize these proteins will help researchers to better understand selenium’s mechanisms of action. 

Additional pilot human studies testing proper doses and formulation and smaller trials in specific populations combined with further preclinical studies on selenium’s mechanisms of action are necessary before the undertaking of any new large phase III clinical trials [83]. 

Above all, it is imperative to determine the subpopulations that can benefit from nutritional intervention. Mixed results from clinical and epidemiological studies underscore the difficulty in making nutritional recommendations for cancer prevention to the population as a whole and highlight the need for studies using subgroups at greater risk [84]. Evidence indicates that those with low baseline selenium status or those that live in selenium-deficient regions represent the optimal cohort for studying cancer prevention by selenium (Table 4). Further refinement of eligibility or stratification criteria by age or selenoprotein genotype may also be useful.

**Table 4:** Possible explanations for null findings in SELECT with selenium.

**Agent**	Optimal dose range for selenium has not been established.
	l-selenomethionine may not be most active formulation of selenium.
	A better understanding of selenium biology is necessary.
**Cohort**	Baseline selenium status of participants was too high for the men to receive additional benefit from selenium supplementation.
	Genotype of various selenoproteins of the cohort should be taken into consideration.
	Intervening in older men may miss a critical window for preventive activities of selenium.
**Design**	Possible lag to effect may have occurred.
	Subgroup analyses were not addressed.
